# *In vitro* gas and methane production of some common feedstuffs used for dairy rations in Vietnam and Thailand

**DOI:** 10.5713/ab.23.0058

**Published:** 2023-08-23

**Authors:** N. T. D. Huyen, J. Th. Schonewille, W. F. Pellikaan, N. X. Trach, W. H. Hendriks

**Affiliations:** 1 Animal Nutrition Group, Department of Animal Sciences, Wageningen University and Research, PO Box 338, 6700 AH Wageningen, The Netherlands; 2 Department of Animal Production, Faculty of Animal Science, Vietnam National University of Agriculture, Trau Quy, Gia Lam, 12406, Hanoi, Vietnam; 3 Department of Population Health Sciences, Faculty of Veterinary Medicine, Utrecht University, PO Box 80163, 3508 TD Utrecht, The Netherlands

**Keywords:** *In vitro* Gas Production, Methane Production, Nutritive Values, Ruminants, Tropical Feedstuffs, Volatile Fatty Acids

## Abstract

**Objective:**

This study determined fermentation characteristics of commonly used feedstuffs, especially tropical roughages, for dairy cattle in Southeast Asia. This information is considered relevant in the context of the observed low milk fat content and milk production in Southeast Asia countries.

**Methods:**

A total of 29 feedstuffs commonly used for dairy cattle in Vietnam and Thailand were chemically analysed and subjected to an *in vitro* gas production (GP) test. For 72 h, GP was continuously recorded with fully automated equipment and methane (CH_4_) was measured at 0, 3, 6, 9, 12, 24, 30, 36, 48, 60, and 72 h of incubation. A triphasic, nonlinear, regression procedure was applied to analyse GP profiles while a monophasic model was used to obtain kinetics related to CH_4_ production.

**Results:**

King grass and VA06 showed a high asymptotic GP related to the soluble- and non-soluble fractions (i.e. A1 and A2, respectively) and had the highest acetate to propionate ratio in the incubation fluid. The proportion of CH_4_ produced (% of GP at 72 h) was found to be not different (p>0.05) between the various grasses. Among the selected preserved roughages (n = 6) and whole crops (n = 4), sorghum was found to produce the greatest amount of gas in combination with a relatively low CH4 production.

**Conclusion:**

Grasses belonging to the genus *Pennisetum*, and whole crop sorghum can be considered as suitable ingredients to formulate dairy rations to enhance milk fat content in Vietnam/Thailand.

## INTRODUCTION

In Southeast (SE) Asian countries, the dairy industry produces insufficient milk to support the current demand of these countries [1]. Typically, Thai and Vietnamese dairy cows produce 4,000 to 4,500 kg of milk/lactation with a mean fat content <4% [2]. It is generally accepted that the relatively low milk yield is not caused by genetics as the dairy cows are generally crossbreds between Holstein Friesian (>87.5%) and local breeds [3]. It is, therefore, plausible that the prevailing environmental conditions and management including nutrition, are the main reasons for the level of milk production [4,5].

In SE Asian countries, dairy rations commonly consist of predominantly roughage sources such as crop residues (e.g., rice straw, corn stover) and fully matured grasses. Such roughages are rich in fibre and it is generally accepted that the feeding of fibre-rich roughages in combination with fully matured grasses results in a high milk fat content [6]. From this perspective, and in combination with the genetic background of the dairy cows and low level of milk production, the low milk fat content (i.e., <4%) of Thai and Vietnamese dairy cows is unexpected.

It is well known that acetic acid (Hac) and β-hydroxybutyric acid, but not propionic acid (Hpr) are important precursors of fatty acid synthesis in the mammary gland of dairy cows [7]. Since the aforementioned precursors of milk fat predominantly originate from rumen fermentation, information on the amount and composition of organic matter (OM) that can be fermented in the rumen (i.e., FOM) is of critical importance to modulate milk fat content by means of dietary intervention. Moreover, not only the amount of dietary FOM but also the degradation kinetics of FOM are important in this respect because slowly degrading FOM, typically FOM originating from roughage, shifts rumen fermentation towards greater proportions of Hac. As such, information on the FOM content of feedstuffs, and its rate of fermentation are essential to optimize dairy rations for milk production and composition. In this respect, it is regrettable that the FOM content of feedstuffs is not routinely assessed and is lacking in the major feeding tables in SE Asian countries. This lack of information prompted the current investigation to screen 29 commonly used feedstuffs for dairy nutrition in SE Asia for their FOM contents and their degradation kinetics.

The aim of the current descriptive study was to gain insight into the variation of the FOM characteristics of tropical feedstuffs and to provide a solid basis for understanding the low milk production and milk fat content by dairy cows in SE Asian countries and Thailand and Vietnam in particular.

## MATERIALS AND METHODS

The experimental protocol to obtain rumen fluid samples from rumen cannulated cows was conducted under the Dutch Law on Animal Experimentation and approved (approval nr: 2017.W-0042.003) by the Central Authority for Scientific Procedures on Animals (CCD, The Hague, The Netherlands).

### Sample collection

A total of 29 individual feedstuffs commonly used for dairy cattle feeding in Thailand and Vietnam consisting of fresh grasses, preserved roughages, whole crop ingredients and single concentrates were collected. Common and Latin names of the various feedstuffs are presented in Table 1. The feedstuffs were selected from various locations in Thailand and Vietnam with the assistance of recognized experts in ruminant nutrition (see acknowledgement). All feedstuffs, at the time of collection were used in practical feed formulation of dairy cattle. The samples were oven dried at 60°C until stable weight, stored in plastic bags and transported to Wageningen University & Research (Wageningen, the Netherlands) for analyses.

### Chemical analysis

Upon arrival at Wageningen, feed samples were ground (1-mm screen) using a cross beater mill (Peppink 100 AN, Deventer, The Netherlands) and analysed in duplicate for residual dry matter, crude ash, crude fibre, starch and sugars. Crude protein (CP) was calculated from nitrogen (N×6.25) obtained by the Kjeldahl method [8]. The neutral detergent fibre (NDF; with heat stable α-amylase) was analysed according to Van Soest et al [9], acid detergent fibre (ADF), and acid detergent lignin (ADL) contents were determined according to Van Soest [10]. Volatile fatty acids (VFA) were analyzed using a gas chromatograph (Trace GC Ultra; Thermo Scientific, Milan, Italy) equipped with a flame ionization detector and an Agilent HP-FFAP column (Agilent Tech., Santa Clara, CA, USA; 30 m length, 0.53 mm i.d., 1 μm film) using hydrogen as carrier gas (25 kPa, constant pressure). Isocaproic acid was used as an internal standard.

### *In vitro* gas and CH_4_ production

Total cumulative *in vitro* gas and CH_4_ production were measured using an automated gas recording system [11,12] with gas and CH_4_ being measured over 72 h. Each of the 29 feedstuffs was ground over a 1-mm sieve using a Wiley mill (Peppink 100AN, Olst, The Netherlands). An amount (~0.5 g) of each feedstuff (Table 1) was precisely weighed into 250 mL fermentation bottles (Schott, Mainz, Germany) with each substrate weighed in triplicate bottles for each of two runs. Three bottles of blanks (rumen fluid without feedstuff) were used in each run. Rumen fluid was obtained from three rumen cannulated Holstein-Friesian, non-lactating cows before the morning feeding at the research farm of Wageningen University, the Netherlands. Cows were fed a low quality grass silage (net energy for lactation, 4.37 MJ/kg dry matter [DM]; CP, 99 g/kg DM; NDF, 675 g/kg DM) *ad libitum* and had free access to water. Approximately 250 mL rumen fluid was collected from the front ventral, middle ventral and cranial dorsal sac from each individual cow. The three rumen fluid samples were pooled and filtered through cheesecloth and subsequently mixed (1:2, v/v) with an anaerobic buffer/mineral solution [11] under continuous flushing with CO_2_. Prior to inoculation, fermentation bottles were placed in a shaking water bath kept at 39°C and pre-flushed with CO_2_. Sixty ml of buffered rumen fluid was added to the bottle before being connected to the fully automated gas recording equipment for 72 h at which time the bottles were opened and placed on ice. Six hundred μL of the solution was pipetted into a 1.5 mL Eppendorf tube and 600 μL internal standard solution (isocaproic acid) was added before mixing vigorously. After 5 min centrifuging at 14,000×g, a 0.75 mL sample of the supernatant was taken and mixed with an equal volume (1:1, v/v) of a stock solution composed of 25 mL of 85% (v/v) ortho-phosphoric acid dissolved in 200 mL Millipore water (Merck KGaA, Darmstadt, Germany) and 300 mL of a 4 g/L 4-methylvaleric acid (internal standard) for VFA analysis and stored at −20°C pending analysis.

The degraded OM (OMd) was determined as described by Williams et al [13] and calculated as the difference between incubated and residual OM after 72 h of fermentation.

Precisely 10 μL of the headspace gas was collected from each bottle and directly injected into a gas chromatograph to determine headspace CH_4_ production at 0, 3, 6, 9, 12, 24, 30, 36, 48, 60, and 72 h, as described by Pellikaan et al [12,14]. Briefly, measured CH_4_ production in individual bottles were expressed relative to the maximum production in each bottle and were fitted iteratively with a monophasic model. Methane production at each individual valve opening were then calculated, and cumulative CH_4_ was calculated as the sum of the increase in headspace CH_4_ production between two successive valve openings, and the amount of CH_4_ vented from the bottle.

### Curve fitting and calculations

Gas and CH_4_ production from all samples were corrected for the corresponding production by blank bottles at each time point [11,12]. Using the nonlinear least squares regression procedure of SAS Institute Inc [15], following equation [16] was fitted to the corrected cumulative data:


$$\text{GP}=\sum_{\text{i}=1}^{\text{n}}\frac{\text{Ai}}{1+{\left(\text{Ci}/\text{t}\right)}^{\text{Bi}}}$$

where, gas production (GP) (mL/g OM) is the cumulative produced gas or CH_4_; n = total number of phases, i = number of phases, Ai (mL/g OM) is the estimated asymptotic gas or CH_4_ production in phase i; Bi is a constant determining the switching characteristic of the curve in phase i; Ci (h) is the time at which half of the asymptotic gas or CH_4_ production was reached in phase i and t (h) is the time of incubation.

A tri-phasic model (n = 3) was fitted to the cumulative GP following the procedure as described by Groot et al [16], where phases 1 and 2 are assumed to relate to the fermentation of the soluble and non-soluble fraction, respectively, and phase 3 is assumed to be related to microbial turnover. The time windows related to the asymptotes of GP for phase 1, 2, and 3 (A1, A2, and A3, respectively) were pre-set from 0 to 3, 3 to 20, and 20 to 72 h after the start of incubation of the substrate, respectively to enable the estimation of the various parameters (Bi and Ci). The aforementioned time points were determined by Van Gelder et al [17]. Data on CH_4_ production were fitted according to the above-mentioned model where n = 1. The maximum rate of gas or CH_4_ production (Rmax, mL/g OM/h) was calculated as described by Yang et al [18].

### Calculations and statistical analyses

Total VFA in fermentation fluid at 72 h was calculated as the sum of Hac, Hpr, butyric acid (Hbu), valeric acid (Hva), isobutyric acid (iso-Hbu) and isovaleric acid (iso-Hva). Branched-chain volatile fatty acids (BCVFA) in fermentation fluid were calculated as the sum of iso-Hbu and iso-Hva. The non-glucogenic to glucogenic ratio (NGR) was calculated as follows [19]:


NGR=[Hac+2×(Hbu+iso-Hbu)+Hva+iso-Hva]/[Hpr+Hva+iso-Hbu].

Prior to statistical analysis of the data related to the fresh grasses, triplicate *in vitro* results of each substrate were averaged per run. Effects of genus and species within each genus on model parameters were subjected to analysis of variance using the PROC MIXED procedure [14] using the following model:


$${\text{Y}}_{\text{ijk}}=\mu +{\text{G}}_{\text{i}}+\text{G}{\left(\text{S}\right)}_{\text{ij}}+{\text{R}}_{\text{k}}+{\text{e}}_{\text{ijk}}$$

where, Y_ijk_ = response variable (i.e. GP-72, CH4-72 production, fermentation kinetics parameters), μ is the overall mean, G_i_ is the effect of genus i (i = 1 to 3), S_j(i)_ is the effect of species j (j = 1 to 4) nested within genus i, R_k_ is the random effect of run k (k = 1 to 2) and e_ijk_ is the residual error term. Differences among species within each genus were determined using the least square means procedure and Tukey’s multiple comparisons [14]. Throughout, the level of statistical significance was pre-set at p<0.05 while a trend was declared at 0.05≤p<0.10.

## RESULTS AND DISCUSSION

Due to their impact on the gas and CH_4_ production curves being minor, the values on the switching characteristics of the GP curves related to the soluble fraction (B1), the non-soluble fraction (B2), microbial turnover (B3), and the B value related to the CH_4_ curve are provided in the Tables but are not specifically addressed or discussed. The focus is mainly on data related to the grasses as well as the preserved roughages and whole crops. The data on single concentrates (Supplementary Table S1 and S2) are provided as a relative benchmark for the grasses, the other roughages and future reference.

### Composition of the fresh grasses, preserved roughages and whole crops

Except for the OM contents, and as expected, the selected feedstuffs showed a large variation in chemical composition (Table 1). For all the selected roughages (i.e. fresh grasses, preserved roughages and whole crops), the greatest variation was found in the CP (14 to 264 g/kg DM) and sugar (0 to 327 g/kg DM) contents. The NDF and ADF contents were high in fresh grasses and preserved roughages, and except for alfalfa hay and cassava hay, NDF and ADF values were found to be ≥631 and ≥333 g/kg DM, respectively. Except for the low starch contents of corn silage, rice straw and whole crop sorghum and corn with cob, no starch was found in the other roughages (Table 1). In contrast to starch, sugars were detected in roughages and especially in fresh VA06 grass, cassava hay, whole crop sunflower and sugarcane, high sugar contents (≥128 g/kg of DM) were found.

Except for fresh VA06 grass and rice straw, the reported composition of the feedstuffs fall within ranges previously reports [20,21]. The CP value of fresh VA06 grass found here was somewhat lower than previously reported which may be explained by the low level of N fertilization commonly practised in Vietnam. Although values as high as 75 and 84 g/kg DM have been reported [22,23] the relative high CP content of rice straw (87 g/kg DM) compared to other reports [23,24] may be explained by the harvesting of relatively young paddy rice. The analyses showed that the rice straw also contained some starch which is most likely explained by the presence of paddy rice grains left in the straw after threshing.

As mentioned above, the values related to cell wall constituents (CF, NDF, ADF, and ADL) are very high in fresh grasses. Such high values indicate that the fresh grasses were cut at a mature stage [25]. Indeed, fresh *Pennisetum* grasses are traditionally cut at a height of >200 cm in SE Asian countries to obtain a high DM yield per cut. Harvesting of fresh grasses at an advanced maturity stage also negatively affects the CP content of the grasses [26,27]. To the authors’ knowledge, the chemical composition of green bean shells has not been reported previously. Its relative high CP content indicates that it may have great potential in ruminant nutrition.

### *In vitro* indices of the FOM content of fresh grasses

Variables such as the cumulative *in vitro* gas production at 72 h (GP-72 h), OMd, and total volatile fatty acid (TVFA) were considered as indices of the *in vivo* FOM content of the fresh grasses. Total gas production (GP-72 h) was influenced by genus but not by species within genus (Table 2) and mean values across species within genus (*Brachiaria*, *Panicum*, and *Pennisetum*) were found to be 222, 226, and 245 mL/g OM, respectively. The asymptote GP associated with the soluble fraction (A1) was similar between *Brachiaria* and *Panicum* but greater values were found for *Pennisetum* because of the high A1 value of VA06 grass. This high A1 value can be explained by the high sugar content (i.e., 138 g/kg DM) of VA06 grass (Table 1). The asymptote GP associated with the non-soluble fraction (A2) was lowest in the genus *Brachiaria* and highest in *Pennisetum*. Parameter A2 was significantly influenced by species within the genus *Panicum* but post-hoc probability values on type I error indicated that the difference in A2 values between Guinea grass and TD59 only tended to be different. The asymptote GP associated with microbial turnover (A3) was similar between genera but within *Brachiaria*, the A3 value of especially Para grass was lower compared to the other 3 grass species within this genus. Within *Pennisetum*, the A3 value was highest for Napier grass. The relevancy of A3 values in relation to feed evaluation, however, can be disputed [28]. As such, the asymptote GP values associated with both the soluble and non-soluble fractions of the grasses (i.e. A1 and A2, respectively) *versus* GP-72 values can be considered as more suitable to estimate the FOM value in not only the selected fresh grasses, but also the other selected feedstuffs (Table 1).

The *in vitro* OMd of fresh grasses (Table 3) were affected by genus as well as species within genera. The highest OMd values were found within the genus *Pennisetum*, which is line with the A1 and A2 values of this genus. Within the genus *Pennisetum*, however, the OMd of VA06 grass was lower compared to that of King grass and Napier which does not agree with the corresponding A1 and A2 values. The latter observation extents to the genera *Brachiaria* and *Panicum*, where also a discrepancy between OMd and the corresponding A1 and A2 values was observed. These discrepancies are not easy to explain but it can be speculated that the variation in OMd does not properly reflect the FOM content of the grasses due to the relative long incubation time, i.e. 72 h [28]. The absolute amount of these VFA is of critical importance to determine the absolute amounts (g/d) of milk fat and lactose synthesized in the mammary gland of dairy cows. The total production of VFA produced in the rumen is primarily determined by FOM intake [7]. In contrast to OMd, the TVFA concentration after 72 h of incubation (Table 3) was not affected by genus. The TVFA concentration was found to be affected by species within genera, but significant differences between specific grass within the genus *Brachiaria* could not be identified by Tukey’s test. Under the assumption that the A1+A2 values are the most suitable indicators of the FOM content of the fresh grasses, it can be concluded that King grass and VA06 are the most promising grasses to increase the FOM content of dairy rations in Vietnam and Thailand.

### Degradation rate of OM and VFA profile after fermentation of fresh grasses

Variables such as the half-time of A1 and A2 and the maximum rate of GP related to phase 1 and 2 (i.e. C1, C2, Rmax1, and Rmax2) can be considered to be relevant to provide some insight into the degradation rate of OM. The half time of asymptote GP of the soluble fraction (C1, Table 2) was affected by genera but not by species within genera; the half-time of the maximum rate of GP was reached ~40 min earlier in the genus *Brachiaria* compared to genera *Panicum* and *Pennisetum*. Such a difference in C1 is, however, considered of minor practical interest. The maximum rate of GP related to phase 1 (i.e., Rmax1) was neither affected by genera nor by species within genera. The latter result most likely indicates that the chemical composition of the soluble fraction and their fermentability in fresh grasses is similar between the various grass species. The soluble fraction of tropical fresh grasses typically contains compounds such as sugars and it is generally accepted that sugars are fermented rapidly [29].

The half time of asymptote GP of the insoluble fraction of fresh grasses (C2, Table 2) was influenced by both genera and species within genera. On average, C2 values were ~13% lower in *Pennisetum* compared to *Brachiaria* and *Panicum* species. Within *Pennisetum*, the C2 value of VA06 was lower than Napier grass and tended (p<0.10) to be lower compared to that of King grass. Within the genus *Brachiaria*, the C2 value of Para grass was found to not differ to that of Humidicola with both were lower compared to Mulato II and Signal grass who were not different. The results on the maximum rate of GP of the insoluble fraction (i.e., Rmax2) were found to be influenced by both genera and species within genera. The highest Rmax2 values were found when *Pennisetum* species were incubated, but unlike the corresponding C2 values, the Rmax2 values were found to be not different for the three grass species within the genus *Pennisetum*. Within the genus *Brachiaria*, the highest Rmax2 value was found when Para grass was incubated but the value of Para grass was found to be not different to that of Humidicola. Overall, it can be concluded that the highest fermentation rates were observed when VA06, King grass or Para grass were fermented.

It is well established that especially Hac is an important precursor of fatty acid synthesis in the mammary gland of dairy cows. Proprionic acid, however, is an important precursor for gluconeogenesis and thus of great importance in the intramammary process of lactose synthesis. The amount of lactose produced in the mammary gland of dairy cows is the major determinant in the amount of milk produced. Thus, a high ratio between Hac and Hpr will potentially result in a greater proportion of fat content in milk. Typically, FOM originating from slowly degrading roughage, shifts rumen fermentation towards greater proportions of Hac and will thus potentially result in greater proportions of milk fat. The proportions (Table 3) of Hac, Hpr, BCVFA, and the ratios of Hac to Hpr (A:P) and NGR were affected by both genera and species within genera. Across genera, the highest proportion of Hac was found when *Brachiaria* was incubated while at the same time the lowest proportions of Hpr were found. Consequently, the A:P was found to be the lowest when *Brachiaria* species were incubated. Within the genus *Brachiaria*, the shift in VFA profile towards Hpr was most profound when Signal grass was incubated while in *Panicum* and *Pennisetum*, the incubation of Hamil or VA06, respectively were most influential to lower the A:P. Overall, NGR was similarly affected, because NGR and the A:P were highly correlated (i.e., *r* = 0.96, n = 11, p<0.05) due to the dominance of Hpr in calculating NGR and A:P. The proportion of Hbu was not affected by species within genera but differences tended to be different between the grasses in *Brachiaria*. The proportions of Hva were not affected by genera but were higher after the incubation of Signal grass (*Brachiaria*), Hamil (*Panicum*) and Napier (*Pennisetum*). The proportions of BCVFA were affected by genera and the highest values were found when either *Panicum* or *Pennisetum* was incubated. Within genera, the highest BCVFA values were found for Para grass (*Brachiaria*), Guinea grass and Hamil (*Panicum*) and Napier (*Pennisetum*).

In an attempt to find a relation between parameters describing GP kinetics (i.e., C1, C2, Rmax1, and RMax2) and the profile of VFA, the proportions of Hpr (mol/100 mol VFA), A:P, and NGR were regressed against GP kinetics parameters. Upon visual inspection of the graphs of each regression (data not shown), however, it appeared that the data obtained from Mulato II and Signal grass, both belonging to the genus *Brachiaria*, had a profound effect on all regressions. In these two grasses, the highest Hpr values and thus lowest values on the A:P and NGR were found (Table 5). The observations on the VFA profile after incubation of Mulato II and Signal grass are difficult to explain. Perhaps, these two grasses contain specific compounds in their insoluble fraction that enhance the synthesis of Hpr during rumen fermentation. In case the various regressions were conducted without these two grasses, no significant correlations were found (*r*≤0.10, n = 9, p>0.05) between the proportions of Hpr, A:P, NGR, and GP kinetic parameters. Overall, the data indicate that *in vitro* VFA fermentation values are similar between tropical grasses and that the practical relevance of the differences is considered of minor interest to dairy cow nutrition. The similarity in VFA patterns between the various topical grasses is most likely related to the fact that all grasses were cut at a mature state.

### Methane production during *in vitro* fermentation of fresh grasses

Across the grass species within genera, cumulative CH_4_ production measured after 72 h of incubation (CH_4_-72; Table 2) expressed as mL/g OM were affected by genera and species within genera. *Pennisetum* species had the highest absolute CH_4_-72 values (44.5 mL/g OM), subsequently followed by *Panicum* (39.8 mL/g OM), and *Brachiaria* (37.4 mL/g OM) species. Within the genus *Brachiaria*, the highest CH_4_-72 was found for Para grass, whereas the highest CH_4_-72 values were found in Guinea and Napier grass in *Panicum* and *Pennisetum*, respectively. Statistically significant differences in the asymptote CH_4_ production between specific genera and specific grasses within genera were not detected but across the 11 grass species, the A values correlated well with the cumulative CH_4_ production (i.e., *r* = 0.91, n = 11, p<0.001). As expected, CH_4_-72 (mL/g OM) depended on GP-72, and when CH_4_ production was expressed relative to total GP (CH_4_-:GP-72), the percentage of CH_4_ were not different between genera. Within the genus *Pennisetum*, however, CH_4_-:GP-72 was affected by grass species and the highest value was found for Napier grass. The C value of CH_4_ production tended to be affected by genera but within genera the values were found to be not different between grass species. The Rmax of CH_4_ was influenced by both the three genera and grass species within genera with the highest rate found when *Pennisetum* species were incubated (p<0.05) while within the genus *Brachiaria*, the highest Rmax value was found when Para grass was incubated whereas incubation of Mulato II resulted in the lowest values (p<0.05).

It is generally accepted that a shift in VFA production from Hac to Hpr renders less hydrogen available for the synthesis of CH_4_ [30]. The current data were found to be in line with this mechanism. Across the 11 grass species, CH_4_-:GP-72 positively correlated with the proportion of Hac in TVFA (*r* = 0.74, p = 0.009, n = 11) and negatively with the proportion of Hpr in TVFA (*r* = −0.82, p<0.01, n = 11). This also explains why *Brachiaria* had a higher level of Hpr with lower amount of CH_4_ production compared to *Panicum* and *Pennisetum*. However, as mentioned before the proportions of Hpr for both Mulato II and Signal grass can be considered as outliers and thus may interfere with proper interpretation of the aforementioned correlations. In case the data of Mulato II and Signal grass were omitted, the relative CH_4_ production remained negatively correlated with Hpr (*r* = −0.83, p<0.01, n = 9) and positively with the A:P (*r* = 0.80, p = 0.01, n = 9). The values on CH_4_ kinetics (i.e., C values and Rmax) were found to be unrelated to the proportions of Hac, Hpr as well as A:P and NGR. With the exception of Mulato II and Signal grass, the Hpr values ranged from 19.8 to 22.1 mol/100 mol TVFA whereas the values on A:P ranged from 3.0 to 3.4 mol/mol (Table 3). Thus, despite the fact that the variation in Hpr and the A:P accounted for a significant part the variation in CH_4_ production, the practical relevance of the variation in the proportions of Hpr and A:P can be questioned.

### *In vitro* indices of the FOM content of preserved- and whole crop roughages

The mean value GP-72 h across the 10 feedstuffs of this group was found to be 218 mL/g OM (Table 4). The lowest values on the asymptote GP associated with non-soluble fraction (i.e. A2) was found when cassava top hay and Pangola hay were incubated but the corresponding numerical value of rice straw was only ~10% higher compared to Pangola hay. Such low A2 values might be due to the very low starch content of these feedstuffs. The lowest A1 value was found when corn silage was incubated because of a lack of sugar to enable rapid fermentation in the first three hours. The OMd and TVFA values were found to be lowest in Pangola hay and greatest in sorghum.

### Degradation rate of OM and VFA profile after fermentation of preserved- and whole crop roughages

The *in vitro* gas kinetics of preserved- and whole crop roughages are presented in Table 4. The greatest value on the half time of asymptote GP of the soluble fraction (C1) was found in sugarcane which is most likely explained by the very high sugar content of this feedstuff. A negative correlation in phase 2 was found between the maximum rate of GP of the insoluble fraction (Rmax2) and the corresponding C2 values (*r* = −0.68, p = 0.016, n = 10). This was obvious in rice straw as it had the lowest Rmax2 value and the lowest C2 value.

As can be seen in Table 5, the selected feedstuffs of preserved roughages had relatively similar values of Hac percentage while fairly large differences were observed for the whole crops. Sugarcane had the lowest Hac value and greatest Hpr and produced the lowest NGR and A:P. It should be noted that Hac and A:P are not only a good indicator of milk fat synthesis but also equally affect animal performance, therefore, sunflower could be considered a good feed ingredient to increase the fat percentage of milk because of its high values for Hac and A:P.

To determine the strength of the relationship between GP kinetic values and VFA profiles, linear, single regressions were performed. The C1 values were found to be negatively correlated with Hac, A:P and NGR (*r* = −0.76, −0.74, −0.76, with p = 0.01 for all) while the C1 values were positively correlated with Hpr (r = 0.80, p = 0.01). Thus, the feedstuffs with a higher C1 value are likely less suitable to increase the fat content of milk.

### Methane production during *in vitro* fermentation of preserved- and whole crop roughages

After 72 h incubation, CH_4_ production (mL/g degradable OM) was greatest for sunflower. However, when expressed as percentage of GP-72, production was the greatest for rice straw. Methane proportion was positively correlated with Hac, A:P and NGR (*r* = 0.73, 0.75, 0.75 and p = 0.02, 0.01, 0.01, respectively) while it was negatively correlated with Hpr (*r* = −0.77, p = 0.01, n = 10). Generally, whole crop roughages had higher GP with a lower CH_4_ proportion compared to preserved feeds.

In general, sorghum is the most interesting feedstuff among the preserved- and whole crop roughages when optimizing dairy rations due to its high FOM content (GP, A1, A2, OMd) and TVFA while it had the lowest CH_4_ production when expressed as a % of total GP.

### Final remarks in relation to the single concentrates

Across eight selected feedstuffs of the single concentrate group (Supplementary Table S1 and S2), it was noted that within approximately 20 h of incubation, more than half of the amount of gas was produced. The concentrate feeds were found to lead to numerically greater GP and OMd than other types of feedstuffs, assumingly due to higher contents of soluble carbohydrates, except for rice bran that had the lowest digestibility values among all the concentrate feeds.

Starch rich feedstuffs had high TVFA and Hbu percentage. Among the concentrate feedstuffs, rice bran had the lowest TVFA, whilst the highest molar proportion of Hac was found for green bean shells. The highest value of Hpr was observed for cassava waste whereas the highest values in TVFA, NGR, CH_4_ production in terms of mL/g degradable OM and the highest rate of GP were reported in cassava peeled tuber.

## CONCLUSION

Of the commonly currently used feed ingredients in Vietnam/Thailand, grasses belonging to the genus *Pennisetum*, and whole crop of sorghum can be considered as suitable ingredients for the formulation of dairy rations to enhance milk fat content. The impact of the cutting of grasses at an earlier physiological stage should be further investigated as a potential tool to increase OMd and milk fat content.

## Figures and Tables

**Table 1:** Chemical composition (g/kg dry matter) of the selected feedstuffs commonly used in dairy cow nutrition in Thailand and Vietnam

Feedstuff	Latin name	OM	CP	CF	NDF	ADF	ADL	Sugar	Starch
Fresh grasses									
Napier	*Pennisetum purpureum* Schumach.	828	157	295	631	336	26	40	-
VA06	*Pennisetum purpureum × Pennisetum americanum*	914	35	366	709	415	56	138	-
King grass	*Pennisetum purpureum × Pennisetum glaucum*	856	109	341	700	375	36	31	-
Guinea	*Panicum maximum* Jacq.	851	133	334	702	388	32	15	-
TD58	*Panicum maximum* cv. TD58	940	120	345	732	404	44	56	-
Mombasa	*Panicum maximum* cv. Mombasa	892	98	383	760	435	34	12	-
Hamil	*Panicum maximum* cv. Hamil	918	129	383	748	416	30	39	-
Para grass	*Brachiaria mutica*	868	93	319	676	360	30	50	-
Mulato II	*Brachiaria ruziziensis* (B. ruziziensis × *B. decumbens* × *B. brizantha*)	927	97	324	716	376	36	41	-
Humidicola	*Brachiaria humidicola*	889	109	296	694	333	30	42	-
Signal	*Brachiaria decumbens*	942	91	368	746	409	44	53	-
Preserved roughages									
Pangola hay	*Digitaria eriantha*	950	39	356	779	418	65	90	-
Alfalfa hay	*Medicago sativa L*.	886	193	288	440	330	59	42	-
Cassava top hay	*Manihot esculenta*	947	264	124	339	157	71	145	-
VA06 silage	*P. americanum* × *P. purpureum*	894	121	361	694	415	44	13	-
Corn silage	*Zea mays L*.	929	80	358	728	411	48	0	36
Rice straw	*Oryza sativa L*.	864	87	310	695	393	35	0	20
Whole crops									
Sunflower	*Helianthus annuus L*.	907	175	171	376	283	98	128	-
Sugarcane	*Saccharum officinarum L*.	980	14	244	567	342	27	327	-
Sorghum	*Sorghum bicolor (L.)* Moench OPV88	930	67	240	574	328	35	-	14
Corn with cob	*Zea mays L*.	926	95	300	693	404	35	-	12
Single concentrates									
Palm kernel cake	*Elaeis guineensis*	955	172	179	754	364	117	21	-
Brewer’s grain	*Hordeum vulgare L*.	966	360	133	611	216	39	-	11
Coconut meal	*Cocos nucifera L*.	993	47	296	735	557	114	7	-
Cassava waste	*Manihot esculenta*	979	19	205	344	275	34	-	477
Green bean shells	*Phaseolus vulgaris*	954	136	306	552	466	58	-	76
Rice bran	*Oryza sativa*	873	103	212	397	241	82	-	217
Corn grains	*Zea mays L*.	989	76	24	114	46	3	-	732
Cassava peeled tuber	*Manihot esculenta*	973	21	36	51	46	6	-	797

OM, organic matter; CP, crude protein; CF, crude fibre; NDF, neutral detergent fibre; ADF, acid detergent fibre; ADL, acid detergent lignin; -, not determined.

**Table 2:** *In vitro* 72 h cumulative gas (GP-72) and methane (CH_4_-72) production and associated model parameters of the organic matter (OM) of three genera (G) of fresh grass species (S) commonly used in dairy cattle nutrition in Thailand and Vietnam

Parameter	Brachiaria				Panicum				Pennisetum			SEM	p-values	
	
	Humidicola	Mulato II	Para grass	Signal grass	Guinea grass	Hamil	Mombasa	TD58	King grass	Napier grass	VA06		G	S(G)
GP-72 (mL/g OM)	217.0	223.6	221.0	226.9	232.1	227.6	227.2	215.2	241.0	244.5	250.8	5.54	0.001	0.460
A1 (mL/g OM)	25.4	21.9	31.4	24.7	10.8	17.5	21.2	22.5	24.1^b^	23.6^b^	49.6^a^	3.66	0.001	0.003
A2 (mL/g OM)	126.2	127.3	133.5	131.5	149.9^x^	140.5^xy^	134.7^xy^	128.3^y^	158.3	140.2	147.2	3.88	<0.001	0.026
A3 (mL/g OM)	65.5^ab^	74.5^a^	56.1^b^	70.7^ab^	71.4	69.6	71.3	64.4	58.6^b^	80.7^a^	53.9^b^	2.99	0.131	0.001
B1	1.07	1.13	1.24	1.23	1.90	1.81	1.31	0.93	1.19	1.16	1.68	0.31	0.210	0.184
B2	2.65	2.58	2.62	2.54	2.88^x^	2.67^xy^	2.87^xy^	2.50^y^	2.61	2.62	2.33	0.08	0.012	0.030
B3	3.72	3.42	4.54	3.50	3.99	3.98	3.72	4.13	4.09	3.80	3.95	0.24	0.550	0.112
C1 (h)	1.14	1.09	1.59	1.09	2.76	1.86	1.64	1.22	2.05	1.86	1.95	0.47	0.011	0.112
C2 (h)	9.00^b^	10.50^a^	8.46^b^	10.76^a^	9.70	10.09	9.85	9.34	8.65^ab^	9.02^a^	7.73^b^	0.38	<0.001	<0.001
C3 (h)	29.63^a^	26.88^ab^	26.57^ab^	25.13^b^	27.81	25.57	28.89	25.89	25.39^b^	28.87^a^	25.71^b^	1.38	0.641	0.001
Rmax1 (mL/g OM/h)	19.4	17.7	14.6	14.9	2.9	7.2	34.0	20.1	8.2	10.7	14.8	9.81	0.696	0.474
Rmax2 (mL/g OM/h)	10.75^ab^	9.19^b^	12.07^a^	9.12^b^	12.62^a^	10.79^ab^	11.12^ab^	10.13^b^	13.94	11.85	13.43	0.49	<0.001	0.002
Rmax3 (mL/g OM/h)	2.21^y^	2.58^xy^	2.38^xy^	2.68^x^	2.73	2.88	2.47	2.72	2.49^ab^	2.85^a^	2.22^b^	0.12	0.007	0.003
CH_4_-72 (mL/g OM)	37.8^ab^	34.2^b^	42.1^a^	35.6^b^	45.5^a^	37.5^b^	39.3^b^	36.9^b^	42.7^b^	50.9^a^	39.9^b^	1.80	<0.001	<0.001
CH_4_-:GP-72 (%)	17.63	15.19	18.66	15.53	19.53	16.41	17.04	17.21	17.63^ab^	21.03^a^	15.65^b^	0.79	0.063	0.002
A (mL/g OM)	50.3	43.4	53.5	47.5	53.6	43.9	47.1	44.0	48.7	62.6	47.6	4.41	0.084	0.036
B	1.28	1.54	1.26	1.43	1.69	1.79	1.62	1.56	1.55	1.44	1.26	0.13	0.007	0.292
C (h)	28.85	29.45	24.75	31.97	25.63	21.90	25.99	24.20	19.46	25.56	19.03	3.91	0.059	0.740
Rmax (mL/g OM/h)	1.26^ab^	0.94^b^	1.42^a^	0.95^ab^	1.29	1.39	1.11	1.11	1.54	1.50	1.63	0.08	<0.001	0.018

SEM, standard error of the mean.

A_i_, asymptote of gas or CH_4_ production in phase i (i = 1,2,3 for gas and 1 for CH_4_); B_i_, sharpness of the switching characteristic for the profile in phase i; Ci, incubation time at which half of maximum gas or CH_4_ production has been formed in phase i; Rmaxi, maximum gas production rate in phase i.

a–c Values within row and within genus with different superscripts differ (p<0.05).

x,y Values within row and within genus with different superscripts show a trend to be different (0.05≤p<0.10).

**Table 3:** *In vitro* 72 h organic matter digestibility (OMd) and fermentation end-products of three genera (G) of fresh grass species (S) commonly used in dairy cattle nutrition in Thailand and Vietnam

Genus	Common name	OMd (g/kg OM)	TVFA (mM)	Hac	Hpr	Hbu	Hva	BCVFA	A:P	NGR
	
				% of TVFA					mol/mol	
*Brachiaria*	Humidicola	639	72.9	67.9^a^	20.6^c^	7.9^x^	1.3^b^	2.3^b^	3.3^a^	3.8^a^
	Mulato II	632	72.2	65.5^ab^	23.8^a^	7.3^xy^	1.3^b^	2.1^c^	2.8^c^	3.2^c^
	Para grass	660	76.1	66.7^a^	21.5^b^	7.7^xy^	1.4^b^	2.7^a^	3.1^b^	3.6^b^
	Signal grass	647	76.8	65.1^b^	24.3^a^	6.9^y^	1.6^a^	2.1^c^	2.7^c^	3.1^c^
*Panicum*	Guinea grass	633^ab^	73.5	67.9^a^	20.6^b^	7.3	1.4^b^	2.8^a^	3.3^a^	3.7^a^
	Hamil	650^a^	75.6	66.0^b^	22.1^a^	7.4	1.6^a^	2.9^a^	3.0^b^	3.4^b^
	Mombasa	591^b^	72.0	67.8^a^	21.0^b^	7.3	1.3^b^	2.6^b^	3.2^a^	3.7^a^
	TD58	586^b^	73.2	67.5^ab^	21.1^b^	7.5	1.4^b^	2.5^b^	3.2^a^	3.7^a^
*Pennisetum*	King grass	683^a^	74.5	67.3	21.3^a^	7.6	1.3^b^	2.5^b^	3.2^b^	3.6^b^
	Napier grass	686^a^	73.0	67.9	19.8^b^	7.4	1.6^a^	3.3^a^	3.4^a^	3.8^a^
	VA06	612^b^	75.1	66.4	22.0^a^	8.3	1.2^b^	2.1^c^	3.0^b^	3.6^b^
SEM		9.04	1.22	0.53	0.37	0.21	0.02	0.05	0.08	0.07
p-value										
G		<0.001	0.336	0.001	<0.001	0.089	0.101	<0.001	<0.001	<0.001
S(G)		<0.001	0.017	<0.001	<0.001	0.037	<0.001	<0.001	<0.001	<0.001

TVFA, total volatile fatty acid; Hac, Hbu, Hpr, and Hva = acetic-, butyric-, propionic- and valeric acid, respectively; BCVFA, branched chain volatile fatty acids; A:P, Hac to Hpr ratio; NGR, non-glucogenic to glucogenic ratio; SEM, standard error of the mean.

a–c Values within column and within genus with different superscripts differ (p<0.05).

x,y Values within column and within genus with different superscripts show a trend to be different (0.05≤p<0.10).

**Table 4:** *In vitro* 72 h cumulative gas (GP-72) and methane (CH_4_-72) production and associated model parameters of the organic matter (OM) of preserved- and whole crop roughages commonly used in dairy cattle nutrition in Thailand and Vietnam

Parameter	Preserved roughages						Whole crop roughages			
	
	Alfalfa	Cassava hay	Corn silage	Pangola hay	Rice straw	VA06 silage	Corn	Sorghum	Sugarcane	Sun-flower
GP-72 (mL/g OM)	236.3	165.2	213.4	173.0	210.4	214.6	221.8	282.5	237.6	228.7
A1 (mL/g OM)	38.2	38.4	14.7	32.0	16.8	24.3	32.4	58.5	55.5	48.5
A2 (mL/g OM)	160.4	102.5	129.1	90.1	99.5	136.0	113.0	161.6	137.6	147.6
A3 (mL/g OM)	37.7	24.3	69.6	50.9	94.2	54.4	76.4	62.5	44.5	32.6
B1	1.3	2.1	1.9	1.4	2.2	1.0	1.4	1.5	4.9	1.9
B2	2.8	2.7	2.9	2.2	2.7	3.0	2.6	2.3	1.7	3.1
B3	4.6	4.6	3.8	3.8	3.6	4.0	4.2	3.7	4.1	4.2
C1 (h)	1.5	1.7	1.6	1.7	1.4	1.1	1.0	1.6	3.2	1.4
C2 (h)	8.1	7.4	10.5	8.1	11.4	9.1	9.4	8.0	5.4	7.4
C3 (h)	23.0	24.9	27.8	28.3	31.3	27.3	29.0	25.0	23.3	27.6
Rmax1 (mL/g OM/h)	29.9	15.3	7.1	12.9	10.3	33.0	21.7	37.0	21.9	22.5
Rmax2 (mL/g OM/h)	15.8	10.7	10.2	7.6	6.9	12.5	9.0	14.0	15.9	17.1
Rmax3 (mL/g OM/h)	1.9	1.1	2.6	1.8	2.9	2.1	2.9	2.5	2.0	1.3
CH_4_-72 (mL/g OM)	40.9	30.3	37.5	33.4	41.3	36.2	42.4	40.0	33.7	43.2
CH_4_-:GP-72 (%)	17.3	18.4	17.6	18.6	19.5	17.1	18.8	14.3	14.3	18.4
A (mL/g OM)	44.3	36.8	43.8	47.5	58.8	41.9	56.5	46.6	43.2	52.4
B	1.8	1.2	1.5	1.1	1.3	1.5	1.3	1.5	1.1	1.3
C (h)	16.8	20.4	21.8	32.0	37.2	20.9	28.7	21.0	21.7	21.3
Rmax (mL/g OM/h)	1.7	1.2	1.2	1.3	1.0	1.2	1.3	1.4	1.6	1.6
Tmax (h)	7.9	2.9	7.7	1.1	8.4	7.2	4.9	7.2	1.3	4.0

A_i_, asymptote of gas or CH_4_ production in phase i (i = 1,2,3 for gas and 1 for CH_4_); B_i_, sharpness of the switching characteristic for the profile in phase i; Ci, incubation time at which half of maximum gas or CH_4_ production has been formed in phase i; Rmaxi, maximum gas production rate in phase I; Tmax, time occurrence of Rmax.

**Table 5:** *In vitro* 72 h organic matter digestibility (OMd) and fermentation end-products of preserved roughages, and whole crops commonly used in dairy cattle nutrition in Thailand and Vietnam

Item	OMd (g/kg OM)	TVFA (mM)	Hac	Hpr	Hbu	Hva	BCVFA	A:P	NGR
	
			% of TVFA					mol/mol	
Preserved roughages									
Alfalfa	653	76.8	67.5	19.8	7.2	1.8	3.7	3.4	3.7
Cassava hay	515	68.7	67.2	20.9	7.8	1.5	2.6	3.2	3.7
Corn silage	567	73.0	66.4	21.6	8.1	1.4	2.5	3.1	3.6
Pangola hay	463	66.1	67.9	20.6	7.9	1.4	2.2	3.3	3.8
Rice straw	572	69.4	67.6	20.6	7.5	1.5	2.8	3.3	3.7
VA06 silage	623	73.8	65.5	23.0	7.4	1.5	2.6	2.8	3.3
Whole-crops									
Corn	595	77.7	68.4	19.2	8.8	1.3	2.3	3.6	4.2
Sorghum	734	83.3	66.5	22.3	7.9	1.3	2.0	3.0	3.5
Sugarcane	586	77.6	62.3	26.9	7.8	1.2	1.8	2.3	2.8
Sunflower	668	75.3	69.7	19.5	6.9	1.4	2.5	3.6	4.0

TVFA, total volatile fatty acid; Hac, Hbu, Hpr and Hva = acetic-, butyric-, propionic- and valeric acid, respectively; BCVFA, branched chain volatile fatty acids; A:P, Hac to Hpr ratio; NGR, non-glucogenic to glucogenic ratio.

## SUPPLEMENTARY MATERIAL

**Supplementary Table S1**. *In vitro* 72 h cumulative gas (GP-72) and methane (CH_4_-72) production and associated model parameters of the organic matter (OM) of single concentrates commonly used in dairy cattle nutrition in Thailand and Vietnam

**Supplementary Table S2**. *In vitro* 72 h organic matter digestibility (OMd) and fermentation end-products single concentrates commonly used in dairy cattle nutrition in Thailand and Vietnam
