# Supplementary material for: In vitro gas and methane production of some common feedstuffs used for dairy rations in Vietnam and Thailand
Source: Anim Biosci. 2023 Aug 23;37(3):481–91. doi: 10.5713/ab.23.0058 (PMC10915187; doi:10.5713/ab.23.0058)
Supplement: Supplementary file 1 [file ab-23-0058-Supplementary-Table-S1.pdf]

**Supplementary Table S1.** *In vitro* 72 h cumulative gas (GP-72) and methane ($CH_4$-72) production and associated model parameters of the organic matter (OM) of single concentrates commonly used in dairy cattle nutrition in Thailand and Vietnam.

| Parameter | Brewer's grain | Cassava waste | Coconut meal | Green bean shells | Palm kernel cake | Rice brain | Cassava (peeled tuber) | Corn grain |
|---|---|---|---|---|---|---|---|---|
| GP-72, mL/g OM | 169.5 | 364.5 | 212.1 | 286.7 | 241.8 | 153.1 | 420.3 | 384.8 |
| A1, mL/g OM | 30.0 | 21.5 | 7.1 | 13.2 | 17.8 | 26.7 | 24.4 | 11.3 |
| A2, mL/g OM | 84.4 | 279.5 | 191.9 | 129.3 | 126.7 | 117.5 | 274.9 | 239.1 |
| A3, mL/g OM | 55.2 | 63.5 | 13.1 | 144.2 | 97.3 | 8.8 | 121.0 | 134.4 |
| B1 | 0.95 | 0.93 | 15.1 | 3.06 | 3.26 | 2.41 | 2.32 | 3.71 |
| B2 | 2.57 | 3.39 | 3.58 | 2.55 | 2.63 | 3.08 | 2.97 | 3.98 |
| B3 | 4.24 | 5.36 | 22.70 | 3.45 | 3.77 | 37.25 | 8.10 | 5.22 |
| C1, h | 1.07 | 2.99 | 9.31 | 1.74 | 1.71 | 1.37 | 1.40 | 1.52 |
| C2, h | 7.31 | 8.75 | 9.18 | 12.47 | 13.18 | 9.84 | 13.25 | 12.32 |
| C3, h | 31.92 | 24.00 | 17.20 | 31.11 | 24.91 | 15.12 | 19.84 | 25.39 |
| Rmax1, mL/g OM/ h | 25.0 | 10.8 | 3.4 | 6.5 | 9.9 | 15.3 | 12.8 | 7.5 |
| Rmax2, mL/g OM/ h | 8.70 | 29.66 | 20.25 | 7.78 | 7.35 | 10.25 | 17.46 | 20.57 |
| Rmax3, mL/g OM/ h | 1.93 | 3.63 | 4.18 | 4.33 | 3.95 | 4.90 | 12.45 | 7.13 |
| $CH_4$-72, mL/g OM | 35.1 | 55.0 | 29.6 | 58.7 | 50.8 | 19.1 | 78.3 | 63.6 |
| CH4-:GP-72, % | 20.75 | 15.17 | 13.97 | 19.93 | 20.63 | 12.19 | 18.41 | 16.41 |
| A, mL/g OM | 138.7 | 60.4 | 29.8 | 75.3 | 58.0 | 20.7 | 80.2 | 67.5 |
| B | 0.88 | 1.67 | 3.06 | 1.66 | 1.84 | 1.53 | 2.82 | 2.34 |
| C, h | 266.06 | 17.73 | 10.60 | 32.87 | 24.19 | 14.14 | 17.54 | 20.52 |
| Rmax, mL/g OM/ h | . | 2.09 | 2.40 | 1.41 | 1.51 | 0.91 | 3.68 | 2.32 |
| Tmax, h | . | 7.67 | 8.49 | 14.02 | 12.35 | 4.90 | 13.48 | 13.86 |

$A_i$ = asymptote of gas or $CH_4$ production in phase i (i = 1,2,3 for gas and 1 for $CH_4$); $B_i$ = sharpness of the switching characteristic for the profile in phase i; Ci = incubation time at which half of maximum gas or $CH_4$ production has been formed in phase i; Rmaxi = maximum gas production rate in phase I and Tmax = time occurrence of Rmax.
