# Supplementary material for: In vitro gas and methane production of some common feedstuffs used for dairy rations in Vietnam and Thailand
Source: Anim Biosci. 2023 Aug 23;37(3):481–91. doi: 10.5713/ab.23.0058 (PMC10915187; doi:10.5713/ab.23.0058)
Supplement: Supplementary file 2 [file ab-23-0058-Supplementary-Table-S2.pdf]

Supplementary Table S2. *In vitro* 72 h organic matter digestibility (OMd) and fermentation end-products single concentrates commonly used in dairy cattle nutrition in Thailand and Vietnam.

Item	OMd	TVFA	Hac	Hpr	Hbu	Hva	BCVFA	A:P	NGR
	g/kg OM	mM	% of TVFA					mol/mol	
Brewer's grains	623	74.1	62.5	22.4	6.6	3.2	5.3	2.8	3.0
Cassava waste	843	91.3	62.1	23.7	10.6	1.4	2.3	2.6	3.3
Coconut meal	757	67.2	60.2	23.8	13.4	1.3	1.2	2.5	3.5
Green bean shells	767	88.8	71.3	17.4	7.3	1.3	2.7	4.1	4.5
Palm kernel cake	689	71.8	66.7	17.5	10.8	1.9	3.1	3.8	4.4
Rice bran	490	60.3	60.4	25.5	9.5	1.7	2.9	2.4	2.9
Cassava (peeled)	960	93.2	62.4	16.6	17.3	1.3	2.6	3.8	5.2
Corn grains	938	91.1	56.6	20.2	18.2	1.6	3.4	2.8	4.2

A:P = Hac to Hpr ratio; BCVFA = branched chain volatile fatty acids; Hac, Hpr, Hbu and Hva = acetic-, propionic-, butyric- and valeric acid, respectively; NGR = non-glucogenic to glucogenic ratio; TVFA = total volatile fatty acid.
